# Prevalence of impacted and transmigrated canine teeth in a Cypriote orthodontic population in the Northern Cyprus area

**DOI:** 10.1186/1756-0500-7-346

**Published:** 2014-06-07

**Authors:** Beste Kamiloglu, Umay Kelahmet

**Affiliations:** 1 Consultant Orthodontist DDS PhD, Department of Orthodontics, Faculty of Dentistry, Near East University, Mersin 10, Turkey; 2 Research Assistant DDS, Department of Orthodontics, Faculty of Dentistry, Near East University, Mersin 10, Turkey

**Keywords:** Impacted teeth, Radiographic evaluation, Transmigration, Premolars, Canine, Retrospective evaluation

## Abstract

**Background:**

The aim of this study was two-fold; (1) to evaluate the prevalence and patterns of impacted canines and transmigrated canine teeth, and (2) to evaluate the possible relationships between impacted teeth, malocclusions and systemic conditions in an orthodontic patient population.

**Methods:**

The clinical records and panoramic radiographs of 453 patients [201 (44.3%) male and 252 (55.7%) female] referred to our outpatient clinic between January 2008 and January 2012 were retrospectively evaluated. The number, position, localization (right/left) and transmigration of teeth, as well as sex, age and systemic conditions of patients, were noted. An impacted canine was considered to be transmigrated when at least part of its length had crossed the midline. Complications related to impacted teeth (pain, cystic changes, root resorption or eruption disturbance of adjacent teeth) were also noted. A p-value less than 0.05 was considered statistically significant.

**Results:**

Impacted and transmigrated canine teeth were found in 16 (3.53%) and two (0.44%) patients in the study group, respectively. Root resorption was seen in four teeth adjacent to impacted canines. No statistical difference was found among gender, location, malocclusion and impaction of the teeth (p > 0.05). However, maxillary canine impaction occurred significantly more frequently than mandibular canine impaction (p < 0.05).

**Conclusions:**

The early detection of impacted as well as transmigrated teeth is crucial for successful treatment, therefore demographic studies are important. Although larger samples are required, this study provides a baseline regarding the frequency and type of impacted canines in this particular population.

## Background

Dental anomalies involving alterations in number, size and structure of teeth often present a major challenge for dental practitioners [1]. Undiagnosed and untreated, many of these dental anomalies may ultimately present complex treatment challenges in the areas of endodontics, orthodontics, prosthodontics and restorative dentistry [1]. From a therapeutic point of view, through early recognition of dental anomalies, many complications can be avoided [2,3]. Baccetti [4] reviewed the literature published before 1963, and reported that there is a possibility that tooth number polymorphism in man is not an isolated phenomenon, but bears a fundamental relationship to the size, development and calcification timing of the dentition as a whole. The spectrum of possible associations among tooth anomalies includes multiple missing teeth, impacted teeth, aplasia of upper lateral incisors and intraosseous displacement of maxillary canines [4].

Tooth impaction is a common dental condition frequently reported in the literature [5-8]. It was stated that when a tooth is unerupted more than 1 year after the normal age for eruption, it is then defined as “impacted” [9]. The prevalence of impacted teeth varies according to the population, and is reported to be between 6.9 and 76.6% [5-10]. The most commonly impacted teeth were reported as third molars, maxillary canines, maxillary central incisors and premolars. These variable rates may have occurred using different age groups, sample sizes and selection of patients [11-14].

Canine impaction is relatively common and has been reported extensively in different populations in the literature, ranging from 0.8 − 3.6% of the general population [15-19]. Transmigration, which is defined as migration of an impacted tooth across the midline, is a rarer condition than standard impaction cases [20]. The prevalence of transmigration in different populations and ethnic groups was the subject of several studies, and was reported to be between 0.1 and 0.34% [19,21]. Accordingly, genetic alterations and ethnic differences were reported to be the key factors for the occurrence of dental alterations [22]. Traits that may occur more commonly in certain ethnic groups may be considered to be specific to that population [3].

Although most impacted teeth are asymptomatic, some can cause complications such as pain, infection cysts, tumors, resorption of the adjacent teeth, jaw fractures, malpositioning of the mandibular anterior teeth and marginal bone resorption near the adjacent teeth [5,23].

The aim of this study was to evaluate the prevalence and patterns of impacted canines and transmigrated canine teeth and investigate the associated pathologies and evaluate possible relationships between impacted teeth, malocclusions and systemic conditions in an orthodontic patient population.

## Methods

This was a population-based, retrospective, descriptive study based on the panoramic radiographs and clinical records of 453 patients [201 (44.3%) male and 252 (55.7%) female] ranging in age from 14 to 20 years, who attended our outpatient orthodontic clinic for evaluation of malocclusions from January 2008 to January 2012. The study was approved by the Institutional Review Board. Also, prior to taking any radiographs or performing intra/extra-oral examination, patients gave their informed consent according to the principles of the Helsinki Declaration, including all amendments and revisions. Collected data were only accessible to the researchers. Moreover, all examiners in the study only examined the radiographs and were blinded to any other patient data in the radiographic examination procedure.

The included sample consisted of 453 panoramic radiographs together with the subjects’ patient records. Digital panoramic radiographs were taken with a Planmeca PM 2002 cc Proline (Helsinki, Finland) set at 1.25 magnification as recommended by the manufacturer, with a maximum sensor resolution of 9 line pairs/mm. All radiographs were acquired with a standardized head position, stabilized by head rods. The exposure settings were dependent on the patient, set from 60–66 kVp, 4–8 mA for 18 s for each exposure, with a half-value layer of 2.47 mm of aluminum. All digital images were stored in a computer database using the manufacturer’s software (Dimaxis Pro, version 4.0.5, Planmeca). Each image was magnified to 110% and contrast and brightness were optimized to produce the best image for viewing under standardized conditions. All reviewing processes were performed on a 17-inch flat panel color active matrix TFT medical display (Samsung SyncMaster 920 N, South Korea) with a resolution of 1280 × 1024.

It has been suggested that the following clinical signs might be indicative of canine impaction [24,25]:

1. Delayed eruption of the permanent canine or prolonged retention of the deciduous canine beyond 14–15 years of age,

2. Absence of a normal labial canine bulge,

3. Presence of a palatal bulge, and

4. Delayed eruption, distal tipping or migration (splaying) of the lateral incisor.

An impacted canine was considered transmigrated when at least part of its length had crossed the midline [17,21]. The numbers, positions and locations (right/left) of impacted/transmigrated canine teeth, as well as patient sex, age, retained deciduous canines and any other associated pathologies, were noted after retrospective evaluation of the patients’ general histories, clinical and radiographic records.

The position of maxillary and mandibular impacted canine teeth were classified according to Mupparapu's classification [26] as:

1. Type 1: Canine positioned mesio-angularly across the midline within the jaw bone, labial or lingual to anterior teeth, and the crown portion of the tooth crossing the midline.

2. Type 2: Canine horizontally impacted near the inferior border of the mandible below the apices of the incisors.

3. Type 3: Canine erupting either mesial or distal to the opposite canine.

4. Type 4: Canine horizontally impacted near the inferior border of the mandible below the apices of either premolars or molars on the opposite side.

5. Type 5: Canine positioned vertically in the midline (the long axis of the tooth crossing the midline) irrespective of eruption status.

Two orthodontic consultants evaluated the images independently prior to the investigation. Calibration of the examiners was undertaken until intra-examiner reliability and reproducibility was achieved. Kappa statistics was used for assessing the agreement between observers using the NCSS 2007 statistical software (NCSS and GESS, NCSS, LLC, Kaysville, UT, USA). Inter-examiner discrepancies were solved by consensus and agreement [27]. To assess reliability, all radiographs were re-examined 2 months after the initial examination by these two observers for the reliability of the results. Pearson’s chi-squared test, Fisher’s exact test and Student’s t-test were performed for statistical analysis of differences in age, gender, localization and measurements (p < 0.05).

## Results

Repeated evaluations showed no significant inter- and intra-observer difference (p > 0.05). There were no discordances in detecting the impacted and transmigrated canines between the two reviews. The kappa values for intra-observer as well as inter-observer detection of impacted and transmigrated canine were 1.0. All observers identified the same number and cases of canine impaction and transmigration in this study.

The distributions of impacted and transmigrated canines according to location and gender are shown in Table 1. Sixteen out of 453 (3.53%) patients had impacted canines (with a total of 18 teeth affected). Of all patients, seven (43.75%) were male and nine (56.25%) were female. Impacted canines were in the maxilla in 12 (75%) patients, while four (25%) patients showed mandibular canine impaction. In terms of impacted canine teeth according to gender, males had 1.99% in the mandible and 6.96% in the maxilla (p < 0.05), while the females’ corresponding values were 1.01% and 3.52% (p < 0.05).

**Table 1:** The distribution of canine impaction and transmigration according to location and gender

**Impaction**	**Patients**	**% in sub-groups**	**P value**	**Males (n)**	**P value**	**Females (n)**	**P-value**
Canine Impaction	16/453	8.16		7		9	
Maxillary Canine	12/453	75	**<0.05**	4	**>0.05**	6	**>0.05**
Mandibular Canine	4/453	25		3		3	
Canine Transmigration	2/453	0.44		1		1	
Maxillary Transmigration	2/2	100	**>0.05**	1	**>0.05**	1	**>0.05**
Mandibular Transmigration	0/2	0		0		0	

P value less than 0.05 indicates significance.

**Figure 1:**
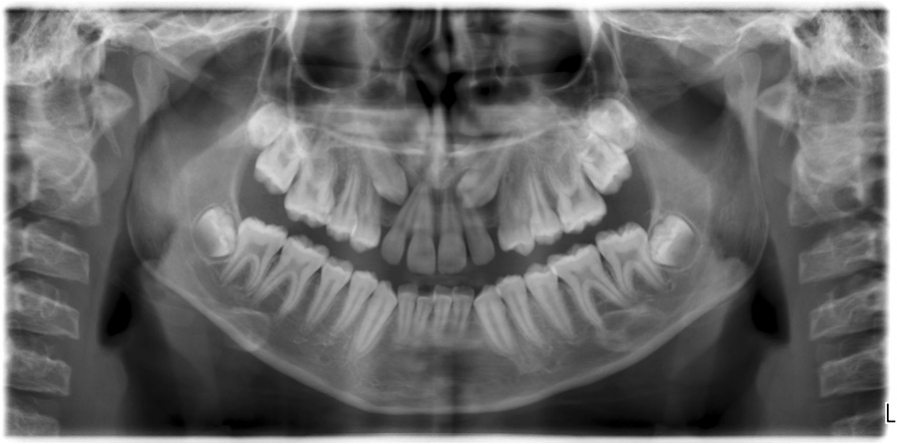
Panoramic image showing bilateral impacted canine in a female patient.

**Figure 2:**
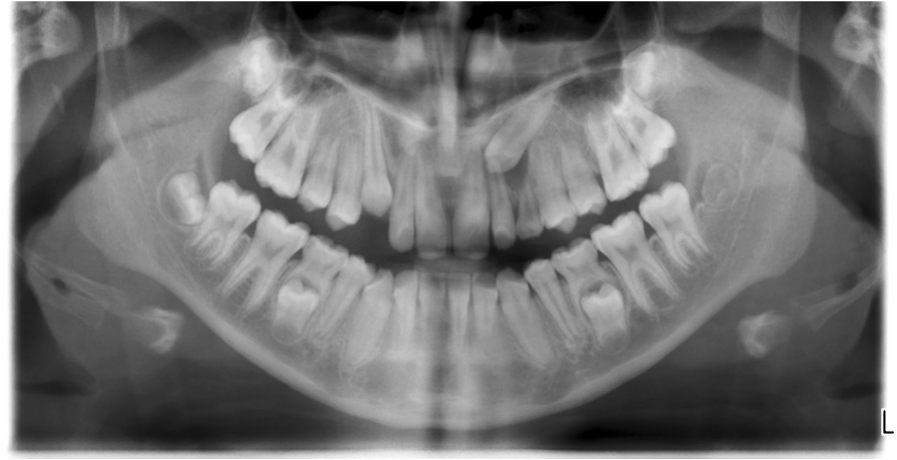
Panoramic image showing unilateral impacted canine in maxilla of a male patient.

There were five right and seven left impacted maxillary canines, while there were two right and two left mandibular impacted canine teeth. Of all patients, two impacted canines were bilateral (Figure 1), whereas 14 were unilaterally impacted teeth (Figure 2). No significant difference was found according to gender for impacted canine teeth (p > 0.05) (Table 2).

**Table 2:** The distribution of number and genders of patients associated with canine impaction

**Gender**	**Number of impacted canines**			**P-value**
	**Unilateral n (%)**	**Bilateral n (%)**	**Total n (%)**	
Female	8 (88.8)	1 (11.9)	9 (100.0)	**>0.05**
Male	6 (88.8)	1 (11.9)	7 (100.0)	
Total	14 (87.5)	52 (12.5)	16 (100.0)	

P value less than 0.05 indicates significance.

For all impacted canines, Type 1 position was the most common. There were no statically significant differences according to gender (p > 0.05). However, we found significant differences for location (p > 0.05), with maxillary canine impaction significantly more frequent than mandibular canine impaction (Table 3).

**Table 3:** The classification of impacted and transmigrated canine teeth in the maxilla and mandible

	**Mandible**		**Maxilla**	
	**Impacted canine**	**Transmigration**	**Impacted canine**	**Transmigration**
**Position**	**n (M/F)**	**n (M/F)**	**n (M/F)**	**n (M/F)**
None	200 / 251	–	193 / 248	–
Type 1	1 / 0	–	3 / 2	1 / 0
Type 2	0 / 1	–	3/ 1	0 / 1
Type 3	–	–	1 / 0	–
Type 4	–	–	0 / 1	–
Type 5	–	–	1 / 0	–
**Total**	**201 / 252**	**–**	**201 / 252**	**1 / 1**

**Figure 3:**
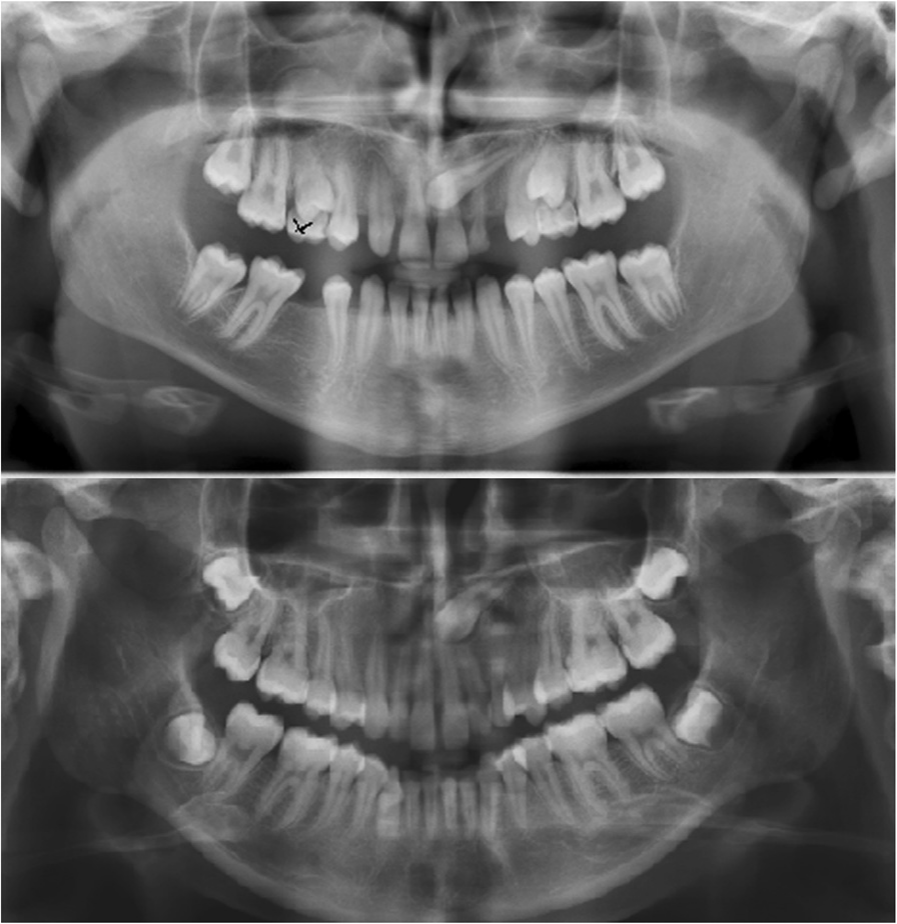
Panoramic images showing transmigrated canine teeth in maxilla.

In all patients, only two (0.44%) transmigrated maxillary canine teeth were found (Figure 3) (Types 1 and 2). No statistically significant difference was found according to gender (p > 0.05). However, these two transmigrated teeth were impacted in the maxilla (Table 3). Associated pathologies were also investigated in this study. Root resorption was seen in four teeth adjacent to impacted canines, all located in the maxilla.

## Discussion

Knowledge of dental anomalies in patients is fundamental for treatment planning [28]. Different prevalence was reported among different ethnic groups [2]. Ethnic background of the sample may result in higher or lower rates of some anomalies [4]. Traits that may occur more commonly in certain ethnic groups may be considered specific to that population [3]. According to Stecker et al. [1], dental practitioners who are aware of ethnic differences in the occurrence of dental anomalies will be more aware in finding them in patients during routine examinations, and may be predictive of normal patterns of tooth development and/or eruption, allowing for prompt clinical intervention to avoid complicating pathology.

Canine impaction is one of the anomalies that should be considered by clinicians in detail. There are various studies in the literature related to impacted and transmigrated teeth [17,18,20,29-37], but only a few comprehensive studies are available. As found in previous reports, canine impaction is more prevalent in the maxilla than the mandible. According to the literature, the prevalence of maxillary canine impaction ranges between 0.8 and 2.8% among different populations. Mandibular canine impaction is relatively rare [16,17,21,38].

Chu et al. [5] investigated the prevalence of impacted teeth and their orientations, but did not mention canine transmigration. Aktan et al. [21] reported canine transmigration together with other impacted teeth, but did not include their orientation. Meanwhile, in their report they excluded third molars similarly to Fardi et al. [38]. As found previously, in this study canine impaction was more prevalent in the maxilla than in the mandible (30 teeth in the maxilla versus seven teeth in the mandible).

In this study, transmigration was also evaluated and a prevalence of 0.44% (2/453) was found. Previous studies indicated a prevalence varying between 0.1 and 4.51%. Shah et al. [10] found eight (0.1%) transmigrated mandibular canines and 4.06% maxillary canine impaction in 7886 individuals. Aktan et al. [21] found a prevalence of 0.34% for transmigrated mandibular canines and 0.14% for maxillary transmigrated canines, while a recent study [37] found a 0.1% prevalence of transmigrated canines in 12,000 patients. However, studies like Zvolanek’s [39] failed to find any cases in 4000 individuals. Our results were in line with previous studies.

Since almost all canine transmigrations are asymptomatic, they are usually diagnosed in routine radiographic assessments [17,20,26,31]. A small number of patients complain of pain, infection, swelling or cyst formation resulting from impacted and transmigrated canines. In this study, root resorption was seen in four teeth adjacent to impacted canines, all located in the maxilla. Bacetti [4] concluded in his review that the existence of associations between different dental anomalies is clinically relevant, as the early diagnosis of one anomaly may indicate an increased risk for others, and reported that the future analysis of a broader spectrum of dental and eruption anomalies in man may reveal further or different patterns or associations. In another study, Sørensen et al. [40] analyzed radiographic evidence of dental deviations with palatally or labially located ectopic canines. Authors of that study recommended that special attention should be given to dental deviations such as invaginations, crown and root deviations including taurodontic molar roots and agenesis as possible risk factors associated with ectopic eruption of maxillary canines, so early identification of patients at risk and appropriate interceptive treatment may reduce ectopic eruption of maxillary canines.

Still, this study has limitations as the sample size was small and the sample population was only representative of the patient pool at the Faculty of Dentistry. Wider population groups should be studied in Cyprus. However, some authors still believe that the prevalence rates of canine impaction may reflect the prevalence rates of these anomalies in the general population.

## Conclusions

The information given in this study could be added to information obtained from other studies on unerupted impacted and transmigrated canine teeth so that a useful clinical database on this particular population could be created to constitute proper plans for managing impacted canines.

## Competing interests

The authors declare that they have no competing interests.

## Authors’ contributions

BK and UK participated in the retrospective evaluation of the data. BK prepared the manuscript. BK and UK are responsible for the literature search and wrote the paper. Both authors read and approved the final manuscript.
